# Effects of jump height on forelimb landing forces in border collies

**DOI:** 10.3389/fvets.2022.1006990

**Published:** 2022-12-21

**Authors:** Joanna Pogue, Chris Zink, Nina R. Kieves

**Affiliations:** ^1^Department of Veterinary Clinical Sciences, The Ohio State University, Columbus, OH, United States; ^2^Zink Integrative Sports Medicine, Ellicott City, MD, United States

**Keywords:** agility, jump height, bar jump, landing force, peak force, peak contact pressure

## Abstract

**Objective:**

The objective of this study was to evaluate the effects of jump height on the landing forces of dogs.

**Animals:**

Client-owned Border Collies experienced in agility competition, *n* = 9.

**Procedures:**

The study involved client owned border collies with the same AKC standard jump height of 20 inches and preferred height of 16 inches. Standard height is based upon the height of the dog at the withers, with preferred height referred to as reduction in jump height by one level due to injury or age. An AKC regulation bar jump was placed over a previously validated pressure sensitive walkway (PSW). The peak force (%BW) and peak contact pressure (kPa) of the leading and trailing forelimbs were evaluated for all dogs.

**Results:**

There was no significant difference in landing force between the two jump heights for either peak force as a percentage of body weight or peak contact pressure when evaluated in both leading and trailing forelimbs.

**Conclusions and clinical relevance:**

Our findings demonstrated no significant difference in active landing forces of peak contact pressure and peak force on the forelimbs of dogs when jumping at a standard jump height vs. a preferred jump height when controlling for velocity in dogs performing a single running bar jump. These results suggest that the recommendation of decreasing jump height for older animals or injured animals may not provide a significant decrease in the impact on the forelimbs. It is likely that other factors contribute to the total forelimb kinematics picture during competition. Veterinarians and trainers should consider additional ways to decrease impact for canine athletes as they recover from injury.

## Introduction

Participation in sporting activities such as agility competitions has become increasingly popular with dog owners. There are over 1 million entries into dog agility competitions sponsored by the American Kennel Club (AKC) yearly. Agility competition is a team sport in which a handler directs a dog through a series of obstacles such as jumps, weave poles, A-frames, and tunnels. The dogs are required to sprint, jump, turn abruptly, balance on narrow plank widths at speed, run over and tip a see-saw, weave back and forth through the gaps between poles set in a straight line, and ascend and descend a steep ramp. Dog and handler teams are rewarded for speed and accuracy.

Given the highly athletic nature of this sport, injuries are common in agility dogs. Recently, it was reported that up to 42% of agility athletes sustain an injury during their career, with the forelimbs commonly affected and reported in up to 60.5% of cases (1–4). The shoulder has been reported to be the most common location of injury for these dogs with injury reports ranging from 12.9 to 30.1% (1–4). Literature also reports that most injuries occurred during obstacle performance during competition, with most injuries (16.9–36.5%) occurring when traversing the bar jumps, which are the most numerous obstacles on any agility course (3, 5).

In both veterinary sports medicine and agility training a common recommendation is to decrease the jump height for dogs that have sustained an injury or are advanced in age. Jump heights used in competitions are standardized based upon the height of the dog at the withers (the dorsal aspect of the scapula). Based on AKC published regulations for bar jumps, there are seven different standard jump heights including 4, 8, 12, 16, 20, and 24 inches. Regulations published for preferred height are defined as jump heights set at 4, 8, 12, 16, and 20 inches based on a drop of a one level from the dog's standard jump height, thus one level would equate to a 4 inch difference in height. The recommendation to decrease jump height is based on the belief that doing so will help to reduce the impact placed on the forelimbs of the dog when landing. When a dog is moved down in jump height by one level, this is termed their “preferred” height by the AKC (6).

Little research has evaluated the kinetics of impact associated with jumps of variable heights used in agility competitions. A limited study of 11 agility dogs evaluating the effect of different jump obstacles on approach speed and landing angle, found that increased vertical forces occurred during the hurdle (vertical) jump compared to the broad (horizontal) jump (5). A recent study assessed the impact of static jumping on landing forces, and found a significant difference in peak vertical forces when landing from a box set at different heights (7). However, no assessment of active landing force over a single bar jump associated with a running jump has been assessed. The effect of varying jump heights has been demonstrated to affect the jump kinetics and kinematics of dogs including approach velocity, jump trajectory, and joint angles as hurdle height increased, but landing forces were not evaluated.

The aim of this study was to evaluate the kinetics of landing on the forelimbs of dogs in a setting simulating training and competing using a single vertical jump of differing heights. We hypothesized that there would be no significant difference in the forces exerted on the forelimbs of the dog upon landing between standard and preferred heights.

## Materials and methods

Healthy Border Collies (*n* = 9) with at least 1 year of experience in agility competition were enrolled in the study with written owner consent and IACUC approval. Border Collies were chosen based on this breed being one of the most common breeds of dogs competing in agility, as well as this breed having a reported increased risk for injury in agility training or competition (1, 2, 4). To control for other variables, all participants had the same AKC standard bar jump height of 20 inches, and preferred height of 16 inches. For this reason, only dogs >18 inches and under 22 inches at the withers were eligible to participate in this study. All dogs were measured from the ground to the dorsal aspect of the scapula (withers) to confirm height and jump category. Prior to participating in the study, all dogs underwent a complete physical examination including orthopedic and neurologic exam, by an experienced veterinarian board-certified in both surgery and sports medicine and rehabilitation (NK). Dogs with orthopedic or neurologic disease were excluded.

**Figure 1:**
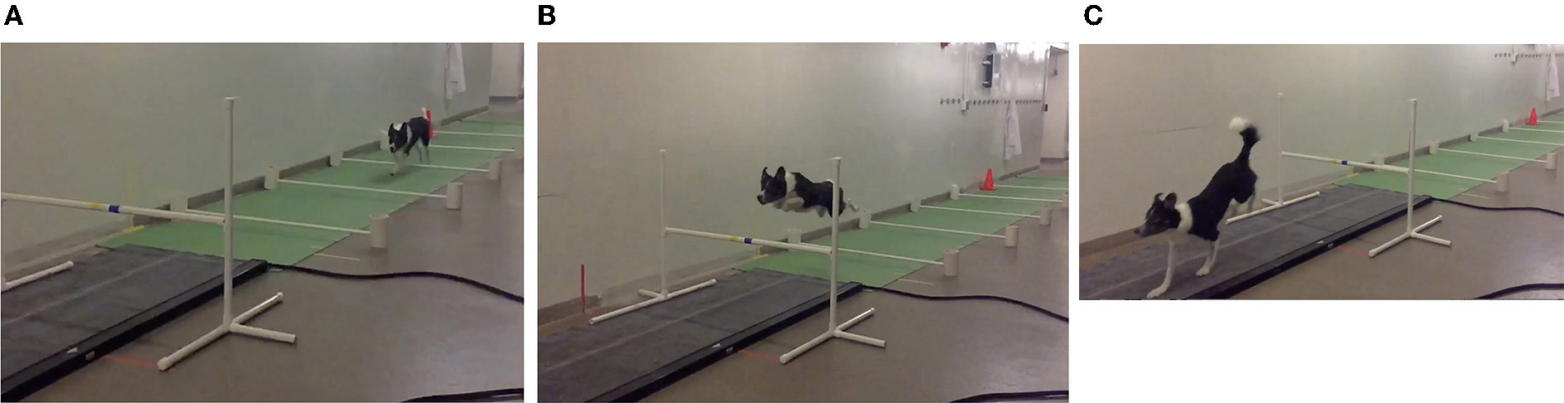
Images of bar jump and PSW set-up. An AKC regulation bar jump (see footnote 1) was placed centrally over a pressure sensitive walkway (PSW) (see footnote 2). Five ground poles spaced 50 inches apart were placed along the runway in front of the bar jump that were three inches above the ground. During the run, the dog jumps between each of the ground poles **(A)**, before taking off between the last ground pole and the regulation bar jump **(B)**, and landing on the PSW **(C)**.

An AKC regulation bar jump^1^ was placed over a previously validated pressure sensitive walkway (PSW) (6).^2^ To consistently regulate speed and velocity of the dog during the approach phase, five ground poles (5′ long, 1″ diameter PVC poles elevated 3″ from the ground) were placed along the runway in front of the bar jump (Figure 1). The distance between the ground poles was set at 50 inches, with the last ground pole placed 60 inches from the bar jump correlating to the preferred jump spot of the dog, with the bar jump situated centrally over the PSW. These distances were determined during a previous pilot study where video recorded trials of participants running over the ground poles at different intervals were evaluated to determine what distance between poles allowed for a consistent, moderate-speed approach of the dogs.

Dogs were allowed to habituate to the room, the walkway and ground poles, and the bar jump. All dogs completed 10 video-recorded trials for both standard and preferred heights. The starting height (standard vs. preferred) was randomly assigned for each dog *via* coin toss. All dogs were given a minimum of 15 mins rest between jump heights. Owners were positioned at the end of the mat facing their dog, with the dog positioned in the typical pre-run agility stance. Trials were considered valid if there was no clear turning of the head from midline, full clearance of bar jump with no contact, and both forelimbs landed completely on the PSW. Data from the first five valid trials for each height was averaged and used for statistical analysis.

The peak force (N), defined as maximum vertical force recorded during landing, and peak contact pressure (kPa), defined as maximum force per unit area upon contact of the forelimb, of the landing forelimbs were evaluated for all dogs. Data were assessed for both the first landing foot (defined as the trailing limb) as well as the second landing foot (or the lead limb), and as an average of both landing feet. Data were normalized to body weight. Peak force as a percentage of body weight (%BW) and peak contact pressure (kPa) measurements between heights were compared using a paired *t*-test (Prism v7.0, GraphPad Software, Inc). Significance was set at *P* < 0.05. The average velocity on approach over the ground poles was calculated for all dogs and averaged using the video-recorded data and standard distance over the ground poles of 50 inches.

## Results

Nine adult Border Collies were enrolled (5 male neutered, 2 female spayed, and 1 each male intact and female intact). Mean weight of the dogs was 15.9 ± 1.9 kg (range 12.7–18.8 kg), and mean age 4.9 ± 2.8 years (range 1–10 years). Mean height of the dogs measured from the ground to the dorsal aspect of the scapula was 53.0 ± 1.4 cm (range 51.2–55.6 cm). All nine dogs jumped at a standard height of 20 inches and preferred height of 16 inches. Mean velocity of the dogs was assessed from video footage. Mean velocity (distance/time) for all dogs was 33.73 ± 5.33 in/s (range 28–43.8 in/s).

**Figure 2:**
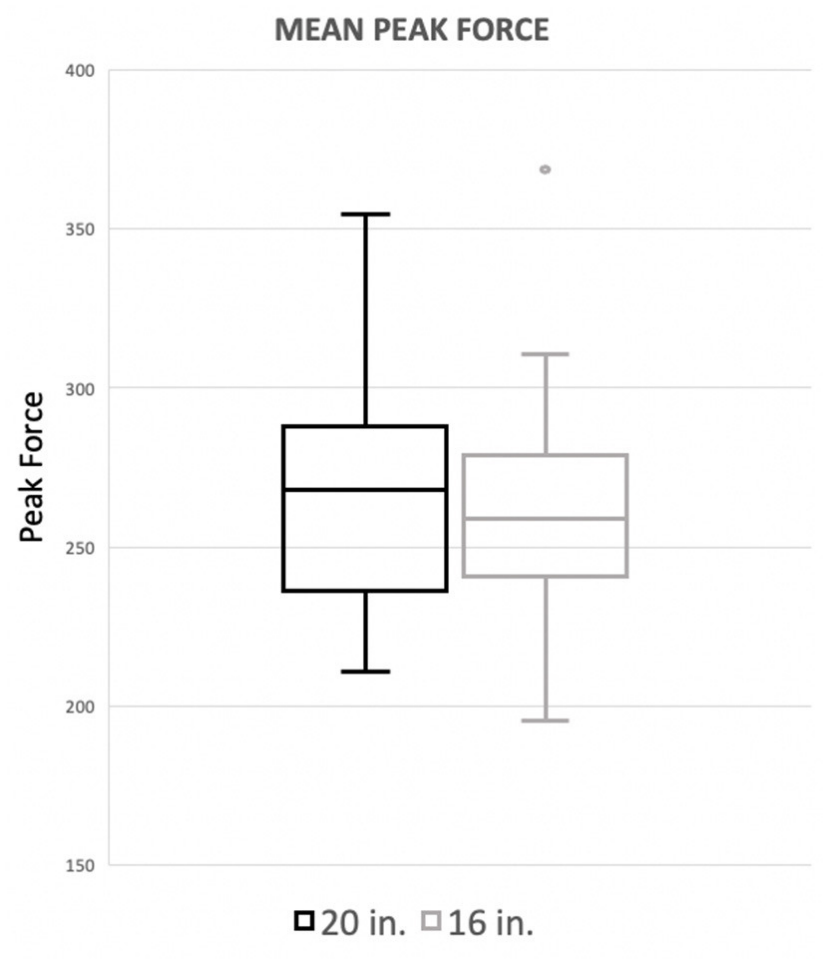
Mean peak force when averaging the forelimbs. There was no significant difference between the standard (20^′′^) or preferred (16^′′^) height for mean peak force of the forelimbs.

**Figure 3:**
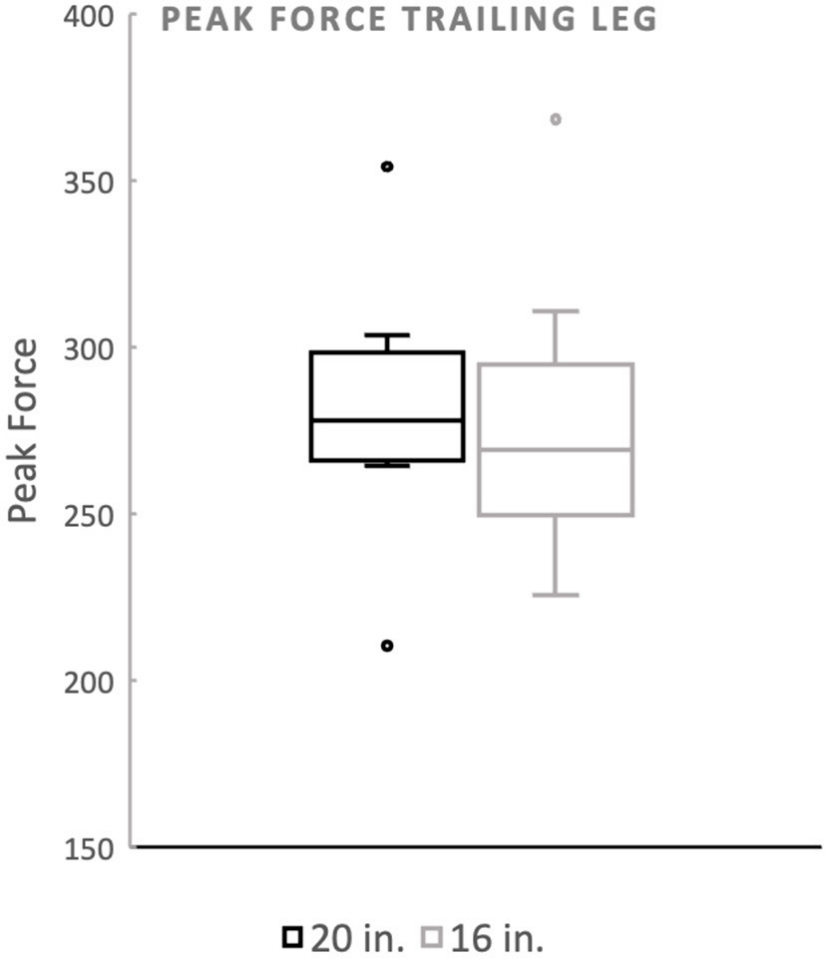
Peak force of the trailing forelimb. There was no significant difference between the standard (20^′′^) or preferred (16^′′^) height for the trailing forelimb. The dots noted outside the box plot are outliers.

**Figure 4:**
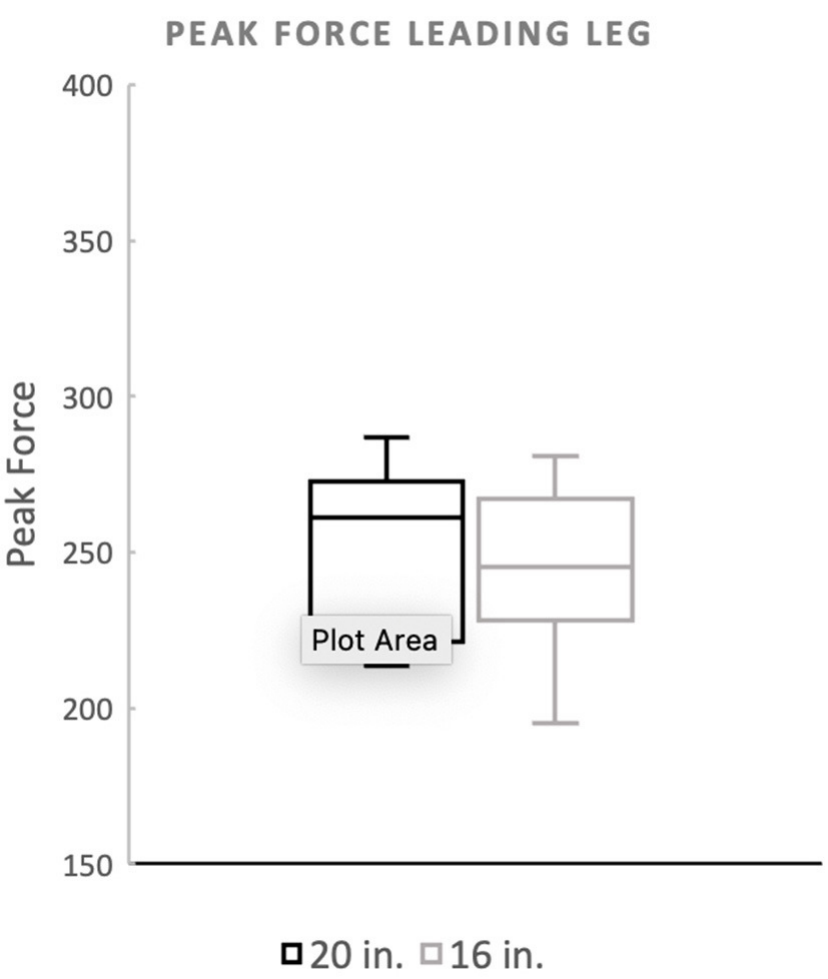
Peak force of the leading forelimb. There was no significant difference between the standard (20^′′^) or preferred (16^′′^) height for the leading forelimb.

**Figure 5:**
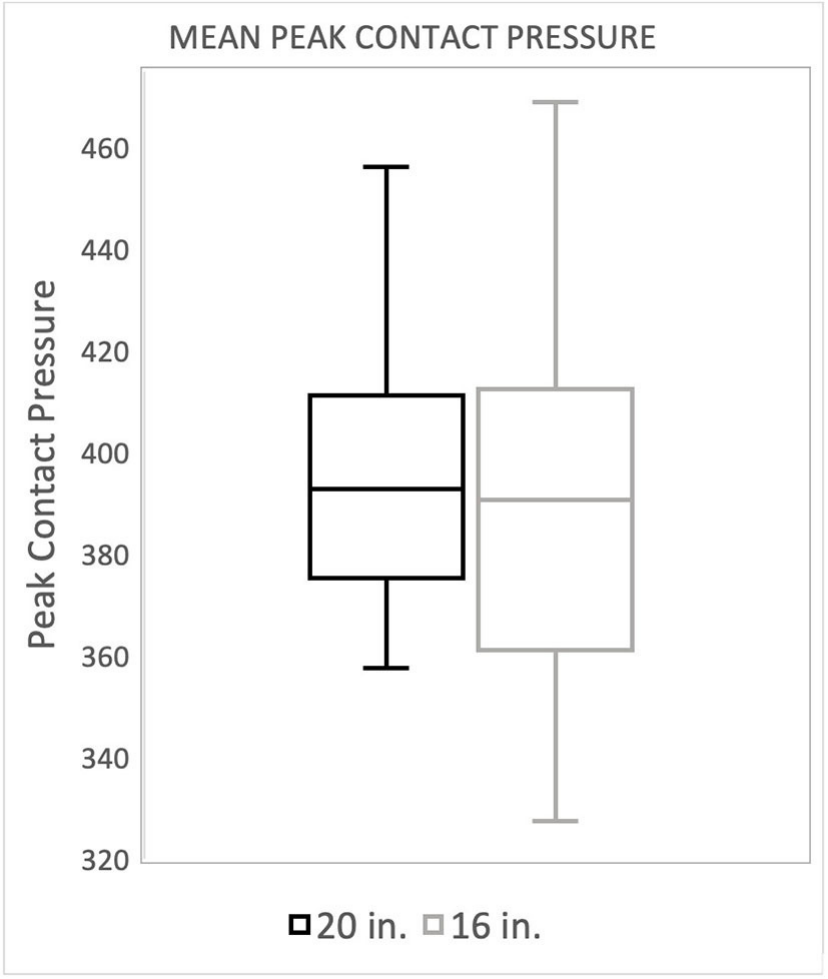
Mean peak contact pressure when averaging the forelimbs. There was no significant difference between the standard (20″) or preferred (16″) height when averaging both peak contact pressure of both forelimbs.

**Figure 6:**
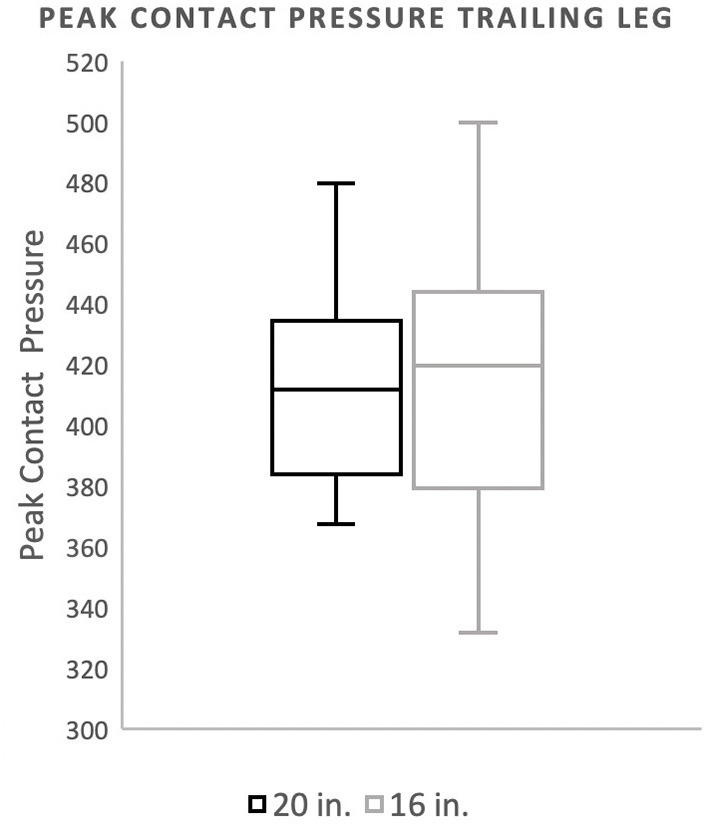
Mean peak contact pressure of the trailing forelimb. There was no significant difference between the standard (20″) or preferred (16″) height for the trailing forelimb.

**Figure 7:**
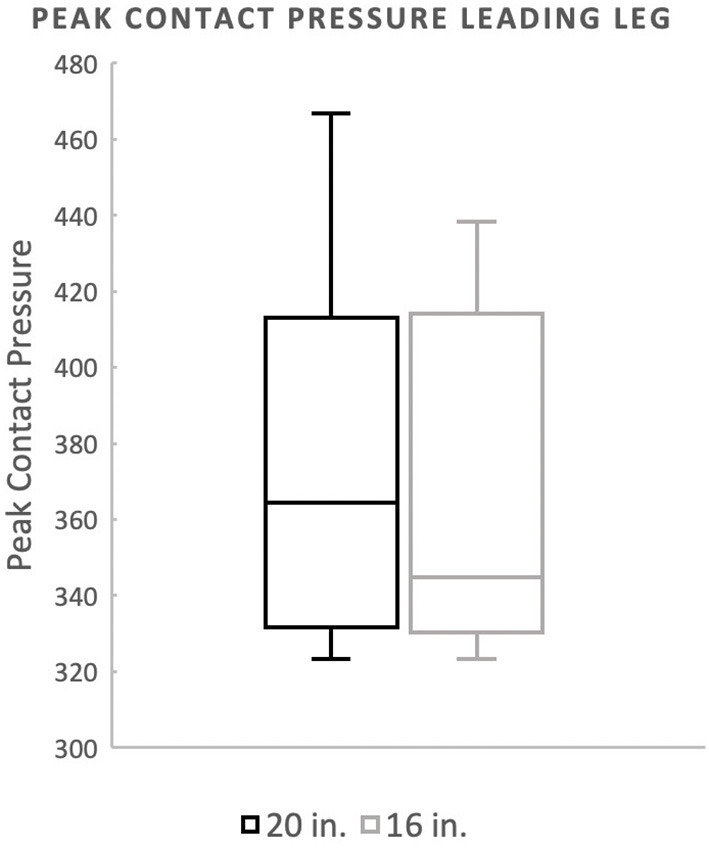
Mean peak contact pressure of the leading forelimb. There was no significant difference between the standard (20″) or preferred (16″) height for the leading forelimb.

There was no significant difference in landing force between the two jump heights for either peak force as a percentage of body weight or peak contact pressure. Mean peak force when averaging the forelimbs was 266.4 (%BW) for the 20^′′^ jump height and 260.9 (%BW) for the preferred jump height (Figure 2). The means of these two groups was not statistically significant (*p* = 0.4228). When evaluating the peak force of the trailing forelimb, the mean peak force was 282.9 (%BW) for the 20^′′^ jump height and 278.1 (%BW) for the preferred jump height (Figure 3). The means of these two groups was not statistically significant (*p* = 0.7081). When evaluating the peak force of the leading forelimb, the mean peak force was 248.3 (%BW) for the 20^′′^ jump height and 241.1 (%BW) for the preferred jump height (Figure 4). The means of these two groups was not statistically significant (*p* = 0.3537). Mean peak contact pressure when averaging the forelimbs was 395.56 kPa for the 20^′′^ jump height and 390.05 kPa for the preferred jump height (Figure 5). The means of these two groups was not statistically significant (*p* = 0.6227). When evaluating the peak contact pressure of the trailing forelimb, the mean peak contact pressure was 406.61 kPa for the 20^′′^ jump height and 377.60 kPa for the preferred jump height (Figure 6). The means of these two groups was not statistically significant (*p* = 0.8890). When evaluating the peak contact pressure of the leading forelimb, the mean peak contact pressure was 393.07 kPa for the 20” jump height and 410.922 kPa for the preferred jump height (Figure 7). The means of these two groups was not statistically significant (*p* = 0.2294).

There was significant variability when evaluating consistency of forelimb lateralization for the leading and trailing forelimb. No dog consistently landed on either the right or left forelimb, whether evaluated at the standard or preferred height.

## Discussion

The purpose of this study was to evaluate the effect of variation in jump height on the landing kinetics of forelimbs in agility dogs when performing a single vertical running bar jump. Our findings demonstrated that there was no significant difference in active landing forces of peak contact pressure and peak force on the forelimbs of dogs when jumping at a standard jump height (20″) vs. a preferred jump height (16″ when controlling for velocity. These results suggest that the recommendation of decreasing jump height for older animals or injured animals when performing the running bar jump may not provide a significant decrease in the impact on the forelimbs of these athletes, though additional studies are needed to confirm this theory.

The jump can be broken down into five phases—approach, take-off, aerial, landing, and departure (8, 9). During these phases, especially during the approach and take-off, the dog must have an appropriate velocity and distance to the obstacle to successfully clear it. The characteristics of the obstacle (including height) can affect these split-second decisions. Studies have demonstrated that as jump height increases there is significant change in joints angles of the forelimb and vertebral column, specifically increased flexion of the radiohumeral and scapulohumeral joints and increased flexion of the base of the neck (7, 10). A significant increase in the height of trajectory and decrease in speed was also found with increasing hurdle height (7). In theory, the longer landing distance for a higher jump height might be secondary to the increased propulsive forces required to clear the jump, resulting in a greater distance between the jump and the landing spot. In support, other studies found that as the height of the obstacle decreases, there is an increase in speed and shallower landing angles of the forelimbs (5, 10). Pfau et al. reported peak vertical force of 4.5 times body weight when landing at a high speed (5). Further, when jump heights were not changed, but distances between jumps was increased, there was an increase in speed coupled with shallower landing angles (11, 12). The change in aerial phase and joint angles due to height of the object would in theory increase the downward velocity and acceleration occurring at landing. While this study attempted to control the approach velocity *via* the use of ground poles, we did not specifically evaluate for changes in velocity and acceleration between standard and preferred heights during the jump trajectory based on limitations with the walkway and cameras, which could impact overall force interpretation. In addition, this study did not evaluate the amount of time under each force. It could be argued that dogs at differing jump heights may have differences in time under pressure, which could ultimately affect force interpretation. Both these concepts should be evaluated in future studies.

The aforementioned studies in conjunction with this study challenge the simple recommendation that reducing bar jump height will decrease injury in agility animals if landing forces have a major impact on injuries. This is the first study to evaluate changes in landing kinetics of peak active force and peak contact pressure during the landing phase when evaluating a single vertical running bar jump. To the authors' knowledge, this is also the first study to regulate approach speed at a consistent velocity when evaluating bar jumps heights in agility dogs. This is an important factor to control in order to obtain meaningful comparative data. This was effected by having the dogs run over ground poles prior to taking off for the bar jump, enabling us to eliminate approach velocity as a factor affecting landing force. Previous studies have not regulated the approach velocity, but rather allowed participants to approach at their own pace. While doing so likely mimics natural adaptations that dogs take when jumping variable heights, it makes it challenging to determine the effect of simply a change in jump height on the kinetics of landing.

The height of the obstacle will not only affect the approach and take-off phases, but also the velocity at impact. Previous studies evaluating jumping down from a stationary position at different heights showed increases in peak vertical ground reaction forces (vGRF) with increase in height (13). In that study, it was noted that the changes in peak vGRF were much smaller than the changes made in the height (13). This correlates with our findings that there is no significant difference in peak contact pressure and peak force on the forelimbs of dogs when jumping at a standard jump height vs. a preferred jump height, and that further investigation into other variables affecting the kinematics of jumping is necessary.

When landing from an obstacle such as the bar jump, the forelimbs are loaded asymmetrically (14). Previous studies of Border Collies noted a shift of weight distribution toward the forelimbs with increasing jump height when landing (5). Dogs generally have an ~60:40 distribution of forelimbs compared to hindlimbs, with border collies specifically having a 58:42 distribution (5, 15–18). The forelimbs have a strut-like action through phases of jumping including take-off and landing (19, 20). During landing, this strut-like action is used to transfer vertical motion into horizontal motion. This results in differences between the leading and trailing forelimbs, with the trailing forelimb being stiffer than the leading forelimb (21). It has been theorized that dogs strike harder with the leading forelimb, but stay longer on the trailing forelimb when landing from a high jump (22). Dogs also primarily brake *via* the trailing forelimb (22). When comparing the lateralization of leading and trailing limb for this study, there was variability identified at both the standard and preferred jump heights. Evaluation of the valid trials runs revealed no consistency in which forelimb was the lead or trail limb for all dogs at both heights. Upon separate evaluation of the both the leading and trailing forelimbs, no significant differences were noted in either peak force or peak contact pressure between jump heights.

Limb stiffness, whether an excess or deficiency, has been associated with injury (23, 24). Excessive stiffness can result in injury to the bone, while a lack of adequate stiffness may result in soft tissue injuries, which are common in agility dogs (1–3, 25). Previous studies in both dogs and horses have shown that the experience of the dog had an impact on jump kinetics (20, 26). Experienced dogs had a higher limb stiffness, decreased limb compression, and higher limb length on landing. They also had a quicker change to propulsion from braking during landing than less experienced dogs. In this study we did not control for experience level in agility among participating dogs. The resultant landing peak force and peak contact pressure did not significantly differ with change in jump height, but it would be prudent to consider evaluating this in more experienced vs. less experienced dogs for better recommendations on the impact this may have on the forelimbs, especially the trailing forelimb.

While in this study, jump height alone had minimal impact on landing force, it is likely that other factors including approach angle, length of aerial phase, landing distance, and other jumps kinematics contribute to the total forelimb kinematics picture during competition. Veterinarians and trainers should consider additional ways to decrease impact for canine athletes as they recover from injury. Dogs with previous agility-related injuries are 100.5 times more likely to experience another injury (27). It is important to note the concept of repetitive stress injury in these dogs. While based on our results there is no significant changes in peak force or peak contact pressure in dogs performing a single running bar jump, the role of repetitive forces on these agility dogs may be more of a significant contributor to injury risk. During the jump, first the shoulder is extending and the elbow is flexing to clear the obstacle and then these joint motions are reversed to prepare for landing (10). Based on a study evaluating muscular activation during jumping, the stride during jumping where the dog lifts off the ground to clear the obstacle and reaches forward with the forelimb to land was consistently the most demanding across forelimb muscles (28). Evaluating these parameters in terms of jump height may be beneficial for future recommendations in reducing bar jump associated injury.

Limitations of this study include the small test population, although it is comparable to other equine and canine studies on jump kinetics (5, 7, 8, 10, 14, 19, 20, 26, 29). In addition, this study also evaluated kinetics of only one breed, Border Collies, and previous studies have shown that breed can affect peak vertical forces as well as the percentages of weight borne on the forelimbs vs. the hind limbs (3, 29). Additional studies should include a wider range of breeds to determine whether there is variability between breeds for various jumping parameters. Because we attempted to control for velocity using set ground poles at specific distances, we chose to standardize dog breed and height to prevent confounding results. Future studies using this method will have to take into consideration optimal pole distance based on dog height and breed. Additionally, this study evaluated the kinetics of landing on the forelimbs of the dog in a setting using a single vertical jump with a straight-line approach. The type of obstacle and the distance between obstacles influences not only the peak vertical force, but also the landing angle, velocity, and jumping distance (5, 10). The agility course and the obstacles included are dependent on the sponsoring organization, venue size/layout, and the judge designing the course. This includes variations in spacing between obstacles, jump heights, and obstacle dimensions. Current research using bar jumps and agility dogs, including this study, evaluated only straight line approaches to the jumps. The control for velocity using ground poles with a set distance has influence on the dog's natural and preferred speed, which in turn can influence other kinetic and kinematic values. As most agility courses consist of multiple obstacles with varying degrees of turns and spacing between them, straight line jumps with set ground speeds do not fully represent jumping in true agility competitions. For example, in a typical standard agility course, at least 65% of the obstacles are jumps, which can be approached from multiple angles and speeds (28). Further studies are necessary to investigate changes in jump kinetics based on jump height in agility dogs approaching the jumps at different angles and preferred speeds. There are also other factors including jump style, fitness level, and handler experience that may have an effect on landing forces. Lastly, this study evaluated force utilizing a validated pressure sensitive walkway system as previously described. When attempting to compare data in the literature regarding magnitude of force values, the use of different gaiting systems should be considered. The magnitude of force values utilizing force plates and pressure sensitive walkways have been correlated, but direct comparisons are difficult to make. Studies comparing these data points in both dog and equine models have shown the peak vertical force was consistently lower for the pressure sensitive walkway when compared to the force plate system, and that these differences were greater when evaluated at a trot compared to a walk (30, 31). The authors note that this is important for future evaluation of canine jump kinetics and subsequent recommendations made from comparing the literature.

This study contributes to the current knowledge of canine jump kinetics that helps to inform decisions for training and competing in dog agility. Our data suggest that the single recommendation of decreasing jump height for older animals or injured animals in agility competitions might not significantly reduce impact on the forelimbs of these athletes. Additional studies will be needed to determine whether recommending a decrease in jump height in combination with other mitigating factors might lessen orthopedic insult to the forelimbs in the Border Collie and other agility athletes.

## Data availability statement

The raw data supporting the conclusions of this article will be made available by the authors, without undue reservation.

## Ethics statement

The animal study was reviewed and approved by IACUC. Written informed consent was obtained from the owners for the participation of their animals in this study.

## Author contributions

JP assisted in study design, data collection, data evaluation, and writing the manuscript. NK and CZ assisted in study design, data evaluation, and writing the manuscript. NK also participated in data collection. CZ participated in study design, statistical analysis, and writing the manuscript. All authors contributed to the article and approved the submitted version.

## Footnotes

^1^“Regulations for Agility Trials” published by The American Kennel Club, amended February 1st, 2016.

^2^HRV WalkwayTM 6 VersaTek System, Tekscan Animal Walkway System, South Boston, MA.
