# Corrigendum: Effects of jump height on forelimb landing forces in border collies

**DOI:** 10.3389/fvets.2023.1233874

**Published:** 2023-08-25

**Authors:** Joanna Pogue, Chris Zink, Nina R. Kieves

**Affiliations:** ^1^Department of Veterinary Clinical Sciences, The Ohio State University, Columbus, OH, United States; ^2^Zink Integrative Sports Medicine, Ellicott City, MD, United States

**Keywords:** agility, jump height, bar jump, landing force, peak force, peak contact pressure

In the published article, there was an error in Figures 2–4 as published. Peak force values were off by a decimal point. The corrected Figures 2–4 and their captions appear below.

**Figure 2:**
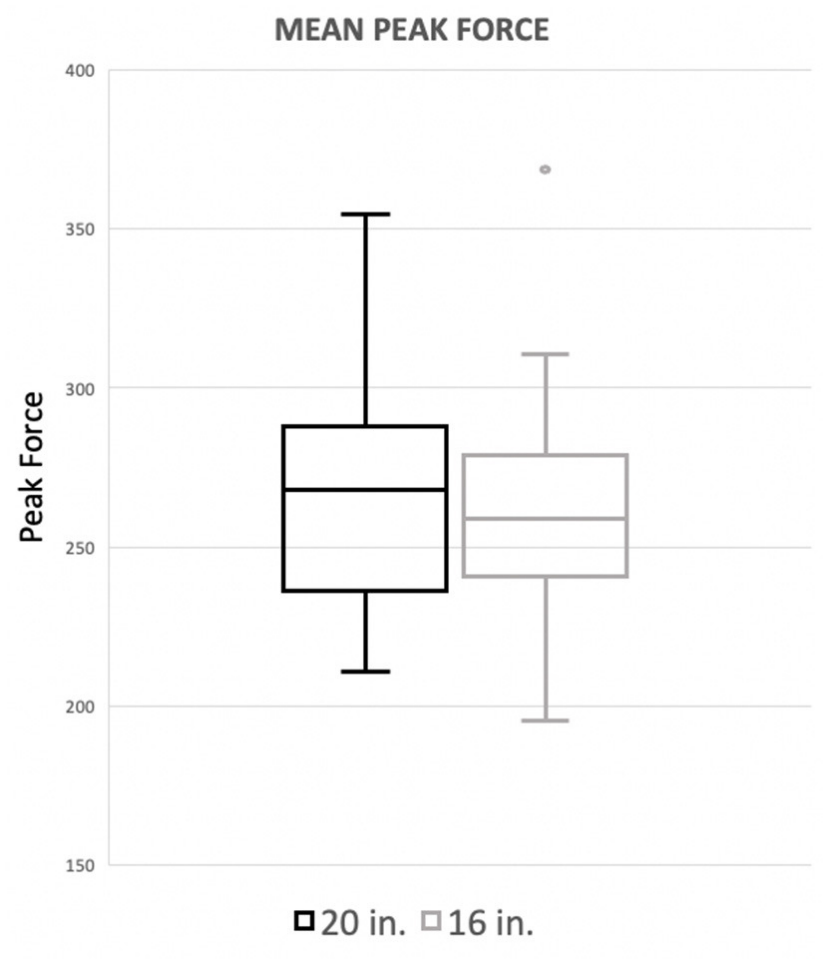
Mean peak force when averaging the forelimbs. There was no significant difference between the standard (20^′′^) or preferred (16^′′^) height for mean peak force of the forelimbs.

**Figure 3:**
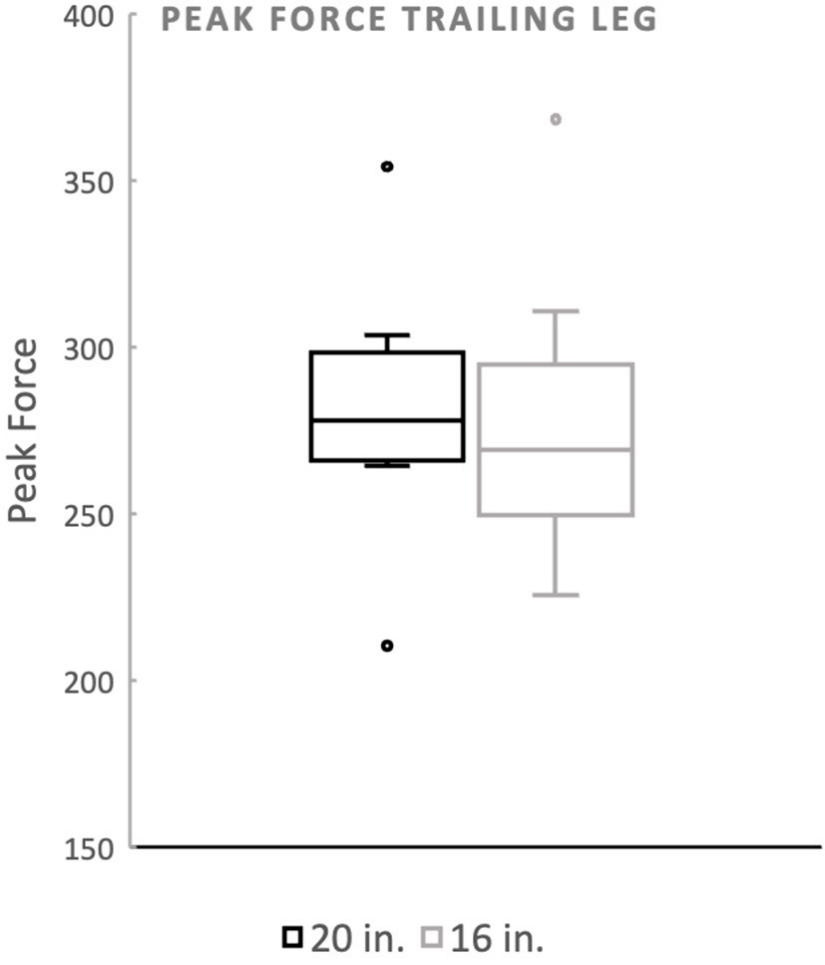
Peak force of the trailing forelimb. There was no significant difference between the standard (20^′′^) or preferred (16^′′^) height for the trailing forelimb. The dots noted outside the box plot are outliers.

**Figure 4:**
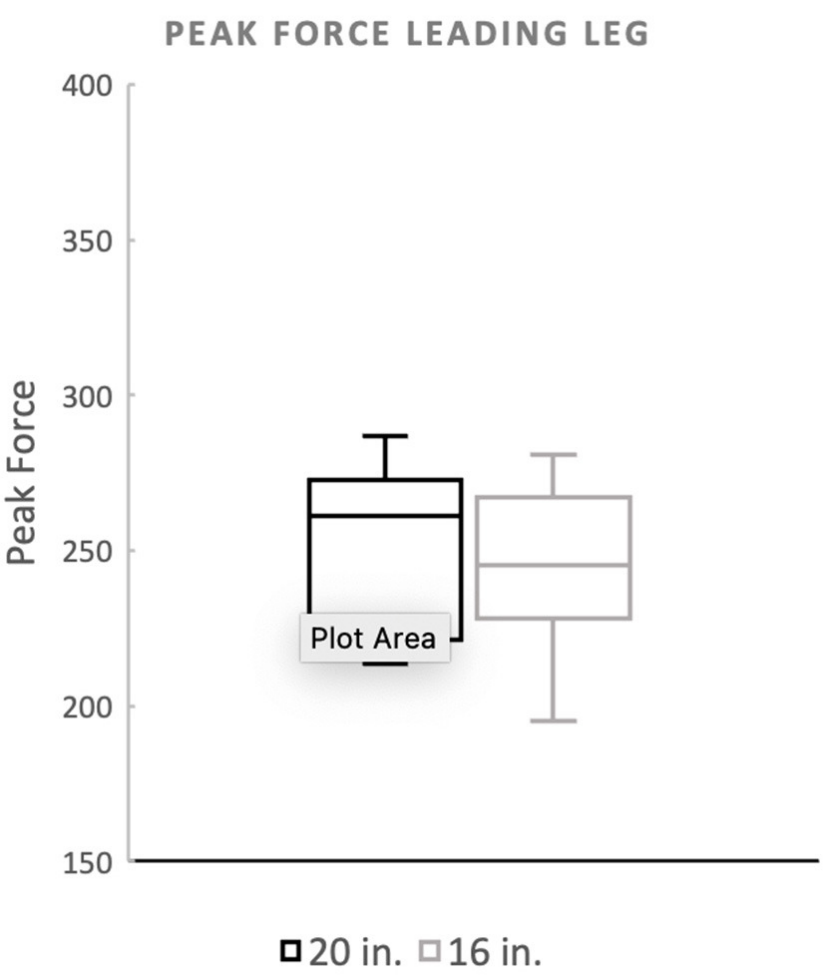
Peak force of the leading forelimb. There was no significant difference between the standard (20^′′^) or preferred (16^′′^) height for the leading forelimb.

In the published article, there was an error. Values for peak force were off by a decimal point.

26.5 should have read 266.4; 26.09 should have read 260.9; 28.29 should have read 282.9; 27.81 should have read 278.1; 24.83 should have read 248.3; and 24.11 should have read 241.1.

A correction has been made to **Results**, paragraph 2. The sentences previously stated:

Mean peak force when averaging the forelimbs was 26.5 (%BW) for the 20^′′^ jump height and 26.09 (%BW) for the preferred jump height (Figure 2). The means of these two groups was not statistically significant (*p* = 0.4228). When evaluating the peak force of the trailing forelimb, the mean peak force was 28.29 (%BW) for the 20^′′^ jump height and 27.81 (%BW) for the preferred jump height (Figure 3). The means of these two groups was not statistically significant (*p* = 0.7081). When evaluating the peak force of the leading forelimb, the mean peak force was 24.83 (%BW) for the 20^′′^ jump height and 24.11 (%BW) for the preferred jump height (Figure 4).

The corrected sentence appears below:

Mean peak force when averaging the forelimbs was 266.4 (%BW) for the 20^′′^ jump height and 260.9 (%BW) for the preferred jump height (Figure 2). The means of these two groups was not statistically significant (*p* = 0.4228). When evaluating the peak force of the trailing forelimb, the mean peak force was 282.9 (%BW) for the 20^′′^ jump height and 278.1 (%BW) for the preferred jump height (Figure 3). The means of these two groups was not statistically significant (*p* = 0.7081). When evaluating the peak force of the leading forelimb, the mean peak force was 248.3 (%BW) for the 20^′′^ jump height and 241.1 (%BW) for the preferred jump height (Figure 4).

The authors apologize for this error and state that this does not change the scientific conclusions of the article in any way. The original article has been updated.

